# Standardizing mercury litterfall and throughfall data for comparative research: Introducing the MFallDa global database

**DOI:** 10.1016/j.dib.2026.113206

**Published:** 2026-08-27

**Authors:** Laura Sereni, Erfan Jahangir, Xinbin Feng, Ivan J. Fernandez, Anne-Hélène Fostier, David Grigal, Antía Gómez-Armesto, Randy Kolka, John Munthe, Tomáš Navrátil, Juan Carlos Nóvoa-Muñoz, Andrea Parente-Sendín, Xabier Pontevedra-Pombal, Emmanoel Vieira Silva-Filho, Hélène Angot

**Affiliations:** a Univ. Grenoble Alpes, CNRS, INRAE, IRD, Grenoble INP, IGE, Grenoble, France; b State Key Laboratory of Environmental Geochemistry, Institute of Geochemistry, Chinese Academy of Sciences, Guiyang, China; c University of Chinese Academy of Sciences, Beijing, China; d School of Forest Resources and Climate Change Institute, University of Maine, USA; e Instituto de Química, Universidade Estadual de Campinas (Unicamp) 13083-862, Campinas, SP, Brazil; f Department of Soil, Water, and Climate University of Minnesota St. Paul, MN 55108, USA; g Universidade de Vigo, Departamento de Bioloxía Vexetal e Ciencia do Solo, Área de Edafoloxía e Química Agrícola, Facultade de Ciencias, Ourense, 32004, Spain; h Instituto de Agroecoloxía e Alimentación (IAA), Universidade de Vigo, Campus Auga, Ourense, 32004, Spain; i USDA Forest Service Northern Research Station, Grand Rapids, MN, USA; j IVL Swedish Environmental Research Institute, PO Box 530 21, 400 14 Göteborg, Sweden; k Institute of Geology of the Czech Academy of Sciences, Rozvojová 269, 165 00 Prague 6, Czech Republic; l GEMAP-CISPAC group, Departamento de Edafoloxía e Química Agrícola, Facultade de Bioloxía, Universidade de Santiago de Compostela, Galiza, Spain; m Department of Geochemistry, Fluminense Federal University (UFF), Outeiro São João Batista, S/N, Centro, Niterói, Rio de Janeiro 24020-141, Brazil

**Keywords:** Vegetation-mediated fluxes, Mercury, Methylmercury, Community data sharing, Global mercury cycle

## Abstract

Vegetation plays a pivotal role in the global mercury (Hg) cycle through the uptake of atmospheric Hg and its subsequent transfer to soils via litterfall (LF), as well as through Hg deposition on leaf surfaces that is washed off by precipitation and throughfall (TF). Yet, global LF and TF fluxes to soils remain poorly constrained, particularly across biomes and climate regions. Moreover, methylmercury (MeHg), that is the most bioavailable and toxic form of Hg, has long been overlooked in terrestrial Hg budgets despite its non-negligible contribution.

This database is the first effort to systematically compile and harmonize all available data on total Hg (THg) and MeHg concentrations and fluxes in LF and TF, ensuring that only datasets with comparable sampling protocols are included. The result is the first global Mercury litter- and throughFall Database (MFallDa, https://doi.org/10.5281/zenodo.21979623). The dataset includes measurements collected between 1987 and 2024 from 147 sites spanning all major biomes, together with metadata on ecosystem type, sampling protocols, precipitation, wet deposition, and Köppen–Geiger climate classification. MFallDa provides an open-access, standardized resource to improve spatially explicit estimates of vegetation-mediated Hg inputs to soils, and refine inter- and intra-site Hg budgets. To facilitate community data sharing and ensure regular updates, a contribution template is provided and the database is hosted on GitHub enabling collaborative input from the global research community. By collecting and standardizing these critical data, MFallDa addresses a key data gap, thereby supporting more robust assessments of vegetation-mediated Hg budgets.

## Graphical abstract

**Figure:**
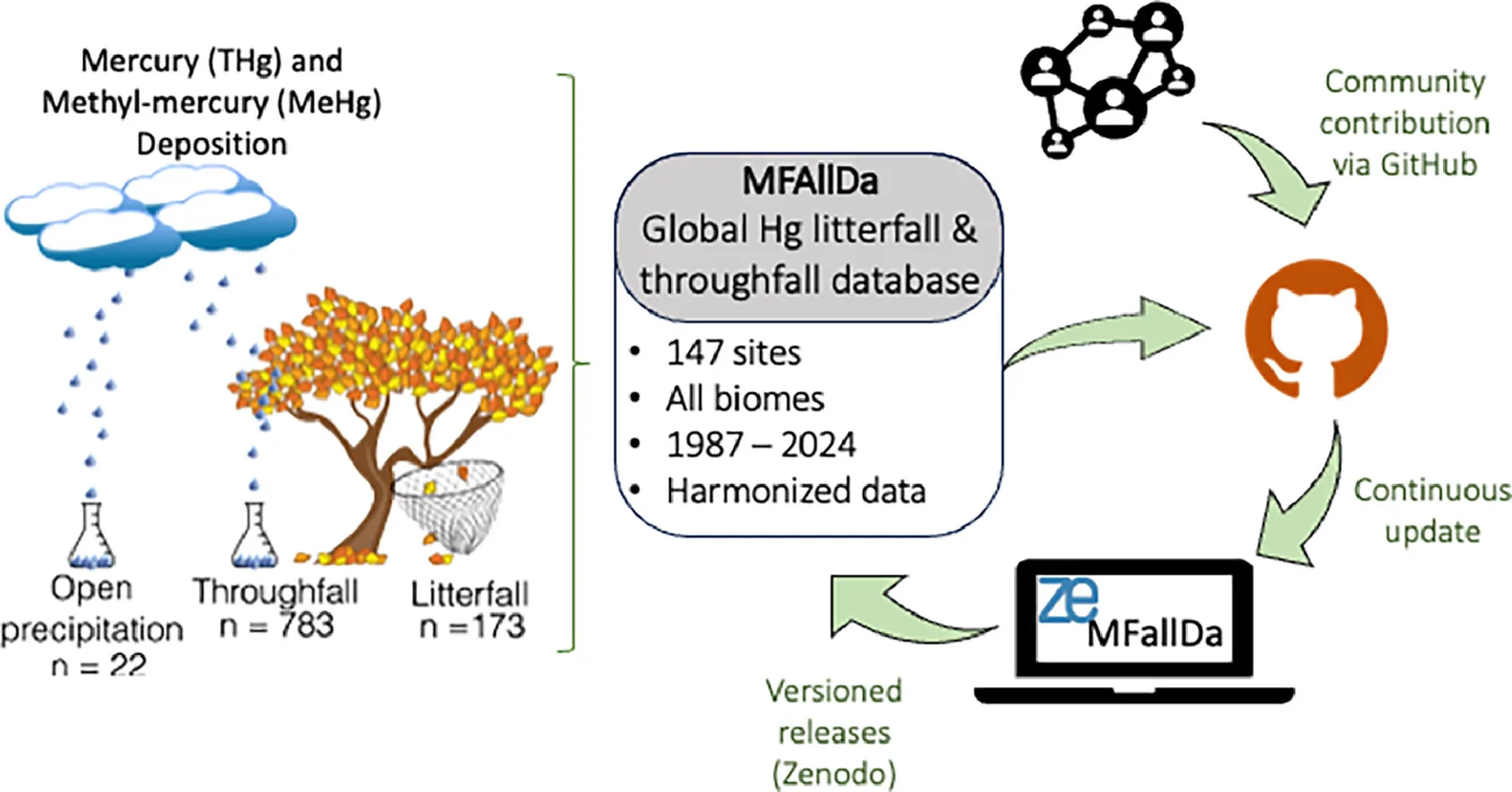

Specifications Table

**Table:** 

Subject	Earth & Environmental Sciences
Specific subject area	Mercury and methylmercury concentrations and fluxes in litterfall and throughfall
Type of data	.csv file (unfiltered dataset with labels from literature search) .csv file (filtered and harmonized dataset) .R file (code producing figures)
Data collection	Data were sourced from peer-reviewed studies measuring mercury or methylmercury (MeHg) in litterfall (collected via traps or baskets) and throughfall. Soil, on-tree plant material samples, or non-annual data were excluded. Metadata –including land cover, geographic coordinates, and Köppen-Geiger climate classification– were added for usability. The dataset comprises two files: (1) literature search results with exclusion criteria, and (2) a harmonized dataset of Hg and MeHg concentrations and fluxes, standardized to consistent units.
Data source location	Data were collected from literature and span all except Oceania and Antarctica continents. Data and code to produce figures are hosted at https://doi.org/10.5281/zenodo.21979622
Data accessibility	Repository name: MFallDa: Data and code accompanying the data paper Data identification number: 10.5281/zenodo.21979622 Direct URL to data: https://doi.org/10.5281/zenodo.21979622
Related research article	none

## 1 Value of the Data

• This dataset provides the first harmonized, global compilation of Hg and MeHg concentrations and fluxes in litterfall and throughfall. Spanning 7 years and 4 continents, it covers 18 of the 30 Köppen-Geiger climate classes and 9 land cover types. As vegetation-mediated Hg fluxes are a major but poorly quantified component of atmosphere-to-soil Hg transfer, this dataset fills a critical gap by offering the most extensive spatial and temporal representation of these fluxes to date.
• The dataset enables researchers to analyze Hg and MeHg fluxes in relation to land cover, climate type, and their interactions. By providing standardized measurements across diverse ecosystems, it allows for direct comparisons and identification of patterns or variability linked to environmental factors, without requiring additional data harmonization.
• Researchers and policymakers can use this spatially explicit dataset to evaluate and refine continental or global Hg models. The inclusion of metadata such as climate classification and land cover facilitates the establishment of regional to global benchmarks, supporting the development of more accurate Hg budgets and the assessment of model performance under varying environmental conditions.
• Designed as a community-driven resource, the dataset supports continuous updates and long-term maintenance. This ensures its ongoing relevance and accuracy, allowing researchers to build on existing data, incorporate new findings, and maintain a dynamic tool for tracking changes in Hg and MeHg fluxes over time.

## 2 Background

Mercury (Hg) is a potent neurotoxin transported globally via the atmosphere and stored for decades to centuries in soils and oceans [2]. Its methylated form, methylmercury (MeHg), is the most bio-available and toxic [3].

Recent research has highlighted the pivotal role of vegetation in mediating Hg exchange between the atmosphere and terrestrial reservoirs. Hg absorbed by vegetation is later transferred to soils through litterfall (LF), while surface-deposited Hg is washed off by precipitation, contributing to throughfall (TF) fluxes [4]. Together, these vegetation-mediated pathways dominate atmospheric Hg transfer to terrestrial ecosystems, accounting for approximately 60–90% of the total atmosphere-to-soil Hg flux over vegetated land [4].

However, current LF and TF estimates are scattered and often heterogeneous, with measurements collected using non-comparable sampling protocols. Therefore, despite numerous studies the identification of reliable spatial, temporal, or global patterns in LF and TF Hg fluxes remains prevented. This limitation is further compounded by the uneven coverage of THg and MeHg data across tissue types.

In this context, a harmonized Mercury litter- and throughFall Database (MFallDa [5]) in which all compiled data follow a clearly documented protocol and use standardized units, thereby facilitating intercomparison across scales, sites, and biomes was constructed.

## 3 Data Description

### 3.1 Files description

This article presents the mercury and methylmercury litterfall and throughfall dataset compiled through a comprehensive literature review, followed by data filtering and harmonization [1].

Two CSV files are provided. The first one, WOS_search.csv, contains the results of the Web Of Science search, indicating for each publication whether data were collected and, if not, a detailed reason for exclusion. The seven columns of this file are described in detail in Table 1.

**Table 1:** Column names and their description for the file WOS_references.csv.

Column name	Content description	Type
Authors	List of authors of the publication	Text
TitleStudy	Title of the publication	Text
Journal	Name of the journal where the publication appeared	Text
PublicationYear	Year of publication	Numeric (YYYY)
DOI	DOI of the publication (starting with “10.”)	Text/Identifier
Keep	Binary value: 1 if data were collected, 0 if not. If 0, the reason for exclusion is provided in the column “ExclusionDetails””	Binary
ExclusionDetails	Details explaining why data were not collected	Text

The second file, MFallDa.csv, contains the collected data, including article sources, location, year of sampling, vegetation type at the sampling site, sampling procedure, tissues types, and THg and MeHg LF and TF concentrations and deposition fluxes. The 34 columns of the MFallDa.csv file are described in detail in Table 2, including units for numerical columns. The column “Location” provides a brief description of the sampling region as indicated in the article, along with the administrative region or province and country. Station names from the National Atmospheric Deposition Program [6] have been homogenized across the studies [[7], [8], [9]]. In addition to THg and MeHg LF and TF concentrations and fluxes, the collected data include long descriptors (“LandCover” and “Vegetation”) and a short identifier (“LandType”) of the vegetation sampled. “LandCover” refers to the biome, “Vegetation” specifies the plant species reported in each study, and “LandType” is a categorical identifier derived from “LandCover” and supplemented with “Vegetation” when necessary. “LandType” follows plant functional types, with categories including: deciduous broadleaved forest, evergreen broadleaved forest, raingreen broadleaved forest, evergreen needleleaved forest, mixed forests, savanna, mangroves, grassland, and one urban for which no better description was available in the original article.

**Table 2:** Column names and format descriptions for the data collected in file MFallDa.csv. The reported number and percentage of data correspond to non-empty entries in the version 1.0 of dataset (https://doi.org/10.5281/zenodo.17816917 [5]). “Individual data” indicates the number of unique values recorded for each variable. NA = not applicable for continuous numerical variables. When submitting new data, bold columns are mandatory and at least one of the italicized columns must be provided**.**

Column name	Content description	Type	Units	Number of data present	Percentage of data present	Number of individual data
AuthorYear	Concatenation of first author name and year of publication; identifier of the publication	Text	-	857	100	71
**TitleStudy**	**Title of the study**	**Text**	**-**	857	100	71
**Location**	**Brief description of the area of sampling with region- administrative region or province- country**	**Text- Text- Text**	**-**	857	100	147
Continent	Continent of sampling (Africa, Asia, Europe, North America, South America, Oceania)	Text	-	857	100	5
**Latitude_N**	**Latitude of sampling**	**Numeric**	**Degree north (decimal)**	857	100	162
**Longitude_E**	**Longitude of sampling**	**Numeric**	**Degree east (decimal)**	857	100	165
**Altitude**	**Above sea level altitude of the sampling site**	**Numeric**	**m**	857	100	154
**StudyYear**	**Sampling period (start year – end year**	**Numeric**	**YYYY - YYYY**	857	100	73
SampleId	Identifier of the measurement; if not provided, constructed using LandType(+_Year) or Tissues(+_Year) in cases where multiple measurement types exist. Each ID corresponds to a single measurement in the study. When study provides only 1 measurement SampleId contains AuthorYear.	Text	-	857	100	857
**LandCover**	**Description of the land cover; level of details varied depending on the article.**	**Text**	**-**	**855**	**99.7**	**35**
LandType	Standardized land cover category;	Categorical	DBF, ENF, RBF, EBF, Grass, Mixed, Mangrove, Savanna, Shrubs, Urban,	854	99.6	10
Vegetation	Plant species reported in the study.	Text	-	741	86.4	61
Climate	Köppen Geiger climate classification	Categorical	Af; Am; Aw; BSh; BWk; BWh; Cfa; Cfb; Csb; Cwa; Cwb; Dfa; Dfb; Dfc; Dwa; Dwb; Dwc;	857	100	18
AnnualPrecip_Volume_mm	Volume of annual precipitation. Most articles don’t disentangle rain- and snow-fall.	Numerical	mm	554	65	155
**MethodLitterfall**	**Litterfall sampling protocol and sampling period**	**Text**		830	96.8	72
**MethodThroughfall**	**Throughfall sampling protocol and sampling period**	**Text**		217	25.3	38
AnnualThroughfall_Volume_mm	Annual throughfall depth	Integer	mm	82	0.09	36
Tissues	Litterfall plant tissues collected; “Total” refers to total collected and unsorted litterfall	Categorical	Total; Needles; Leaves; Fruits; Bark; Misc; Twigs; Lichens	825	96.2	8
*LitterfallHg_MeanConcentration_ngg*	*Mean THg concentration in litterfall, averaged over replicants and sampling period*	*Numerical*	*ng Hg g^−1^ dry weight*	530	60.6	NA
*LitterfallHg_AnnualMeanFlux_ugm2*	*Annual THg flux deposited through litterfall.*	*Numerical*	*µg Hg m^−2^ yr^−1^*	716	83.5	NA
*LitterfallMeHg_MeanConcentration_ngg*	*Mean MeHg concentration in litterfall, averaged over replicants and sampling period*	*Numerical*	*ng MeHg g^−1^ dry weight*	149	17.4	NA
*LitterfallMeHg_AnnualMeanFlux_ugm2yr*	*Annual MeHg flux deposited through litterfall.*	*Numerical*	*µg MeHg m^−2^ yr^−1^*	155	18.1	NA
*LitterfallBiomass_gm2yr*	*Annual biomass flux deposited through litterfall*	*Numerical*	*g dry weight m^−2^ yr^−1^*	437	51.0	NA
*WVMC_TF_Hg_ngL*	*Volume-weighted mean concentration of THg in throughfall (reported explicitly in only two studies)*	*numerical*	*ng Hg L^−1^*	4	0.04	NA
*TF_AnnualMeanConcentration_Hg_ngL*	*Annual mean THg concentration in throughfall*	*numerical*	*ng Hg L^−1^*	86	10	NA
*TF_AnnualMeanConcentration_MeHg_ngL*	*Annual mean MeHg concentration in throughfall*	*Numerical*	*ng MeHg L^−1^*	15	1.7	NA
*TF_AnnualMeanFlux_THg_ugm2yr*	*Annual mean THg flux in throughfall*	*Numerical*	*µg Hg m^−2^ yr^−1^*	157	18.3	NA
*TF_AnnualMeanFlux_MeHg_ugm2yr*	*Annual mean flux of MeHg in throughfall*	*Numerical*	*µg MeHg m^−2^ yr^−1^*	53	6.2	NA
OpenPrecipitation_AnnualMeanConcentration_Hg_ngL	Annual mean THg concentration in open precipitation	Numerical	ng Hg L^−1^	170	19.8	NA
OpenPrecipitation_AnnualMeanFlux_Hg_ugm2yr	Annual mean THg flux in open precipitation	Numerical	µg Hg m^−2^ yr^−1^	509	59.4	NA
OpenPrecipitation_AnnualMeanConcentration_MeHg_ngL	Annual mean MeHg concentration in open precipitation	Numerical	ng MeHg L^−1^	22	2.6	NA
OpenPrecipitation_AnnualMeanFlux_MeHg_ugm2yr	Annual mean MeHg flux in open precipitation	Numerical	µg MeHg m^−2^ yr^−1^	62	7.2	NA
Flag	Additional information about sampling procedure, data quality, or contamination issues	Text		461	53.8	29
Flag_precipitation	Flag column set to “out” for precipitation measurements not taken during the study period *(*e.g.*, multi-year averages or values measured at nearby sites, as reported by the original authors).*			278	32.4	1
DOI	DOI of the article (starting with “10.”)	Text		857	100	71

The collected data were further supplemented with the Köppen-Geiger climate classification, with climate types recorded in the database as follows: tropical rainforest (Af); tropical monsoon (Am); tropical dry savanna (Aw); hot semi-arid climate (BSh); cold desert climate (BWk); hot desert climate (BWh); humid subtropical climate (Cfa); temperate oceanic climate (Cfb); warm summer Mediterranean climate (Csb); monsoon-influenced humid subtropical climate (Cwa); subtropical highland climate (Cwb); hot summer humid continental climate (Dfa); warm summer humid continental climate (Dfb); subarctic climate (Dfc); monsoon-influenced hot summer humid continental climate (Dwa); monsoon-influenced warm summer humid continental climate (Dwb), and monsoon-influenced subarctic climate (Dwc).

The column “Tissues” refers to original tissues when sorted after collection. The following tissue categories were defined: “Total” (unsorted LF, assigned by default when the method does not specify tissues); “Leaves” or “Needles” (for broadleaved and needleleaved trees, respectively); “Bark” (also assigned to the single study reporting “Wood” measurements [10]); “Fruits” (also assigned to cones); “Lichens”; “Twigs” and “Misc” (miscellaneous).

Two additional columns describe the sampling procedures for LF and TF. A “Flag” column provides supplementary information about the study (e.g., “contaminated site”, “urban”) and a “Flag_precip” column indicates annual rainfall depth not measured during the study period (e.g., multi-year averages or values measured at nearby sites, as reported by the original authors).

In line with the open science strategy, the dataset and accompanying documentation are initially stored on GitHub (https://github.com/IGE-mercury/MFallDa), with the first official release archived on Zenodo 10.5281/zenodo.17816917 [5]. The GitHub repository contains two branches: a *main* branch for the stable version of the database which is linked to the official Zenodo release, and a *dev* branch that allows contributions.

Users should download the dataset from the latest Zenodo release (10.5281/zenodo.17816916 [5]) and cite both this paper and the DOI of the database. The GitHub repository is intended solely for submitting new data or updates to the current version of the database. A new release on Zenodo will be created when appropriate, for example when a sufficient amount of new data has been contributed or substantial updates are implemented.

The source code used to produce the dataset description figures is available on GitHub at https://github.com/IGE-mercury/MFallDa_fig/releases/tag/v1.2, with the release archived on Zenodo at https://zenodo.org/records/19696142. A single, stable entry point to both resources (database and code) is available at https://doi.org/10.5281/zenodo.21979622.

### 3.2 Overview of collected data

**Fig. 1:**
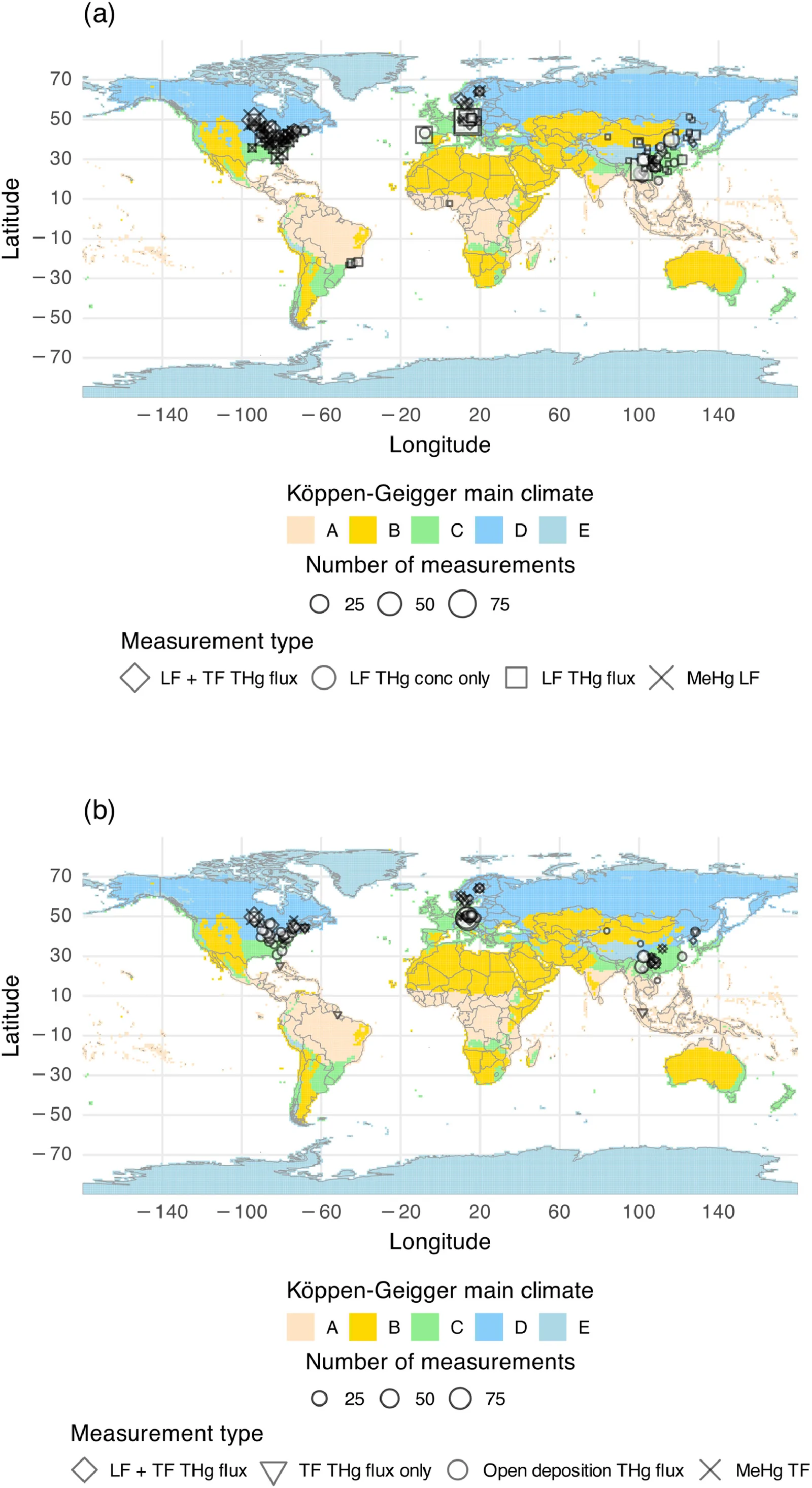
Measurement sites and number of measurements in the collected dataset, shown by measurement type and main climate category (A = Tropical; B = Dry; C = temperate; D = continental; E = polar). (a) Litterfall (LF) measurements; (b) Throughfall (TF) measurements.

**Fig. 2:**
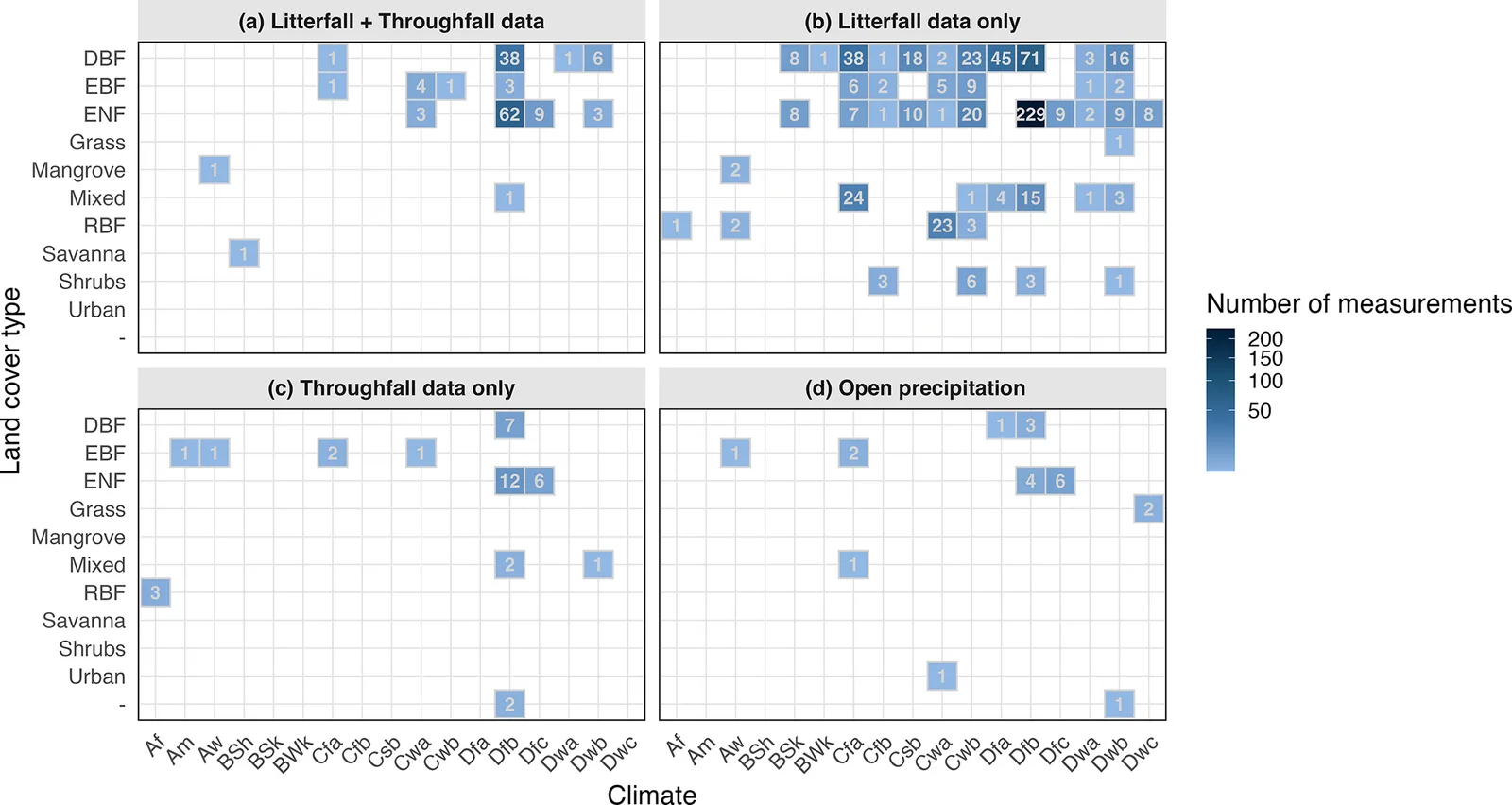
Number of measurements per land-cover type and climate type; cells are left empty where no measurements are available. (a) Number of measurements with both litterfall and throughfall data, (b) Number of litterfall-only data, (c) Number of throughfall-only data, and (d) Number of open precipitation measurements. Land-cover types: DBF = deciduous broadleaved forest, EBF = evergreen broadleaved forest, ENF = evergreen needleleaved forests, Grass = grassland, Mixed = Mixed forest, RBF = raingreen broadleaved forest, Savanna = savanna, Shrubs = shrubs, and Urban = urban forest. Climate types: Af = tropical rainforest; Am = tropical monsoon; Aw = tropical savanna; BSh = hot semi-arid climate; BWk = cold desert climate; BWh = hot desert climate; Cfa = humid subtropical climate; Cfb = temperate oceanic climate; Cs = warm summer Mediterranean climate; Cwa = monsoon-influenced humid subtropical climate; Cwb = subtropical highland climate; Dfa = hot summer humid continental climate; Dfb = warm summer humid continental climate; Dfc = subarctic climate; Dwa = monsoon-influenced hot summer humid continental climate; Dwb = monsoon-influenced warm summer humid continental climate, and Dwc = monsoon-influenced subarctic climate (Dwc).

Collected data span the period from 1987 to 2024, with 16 studies based on samples collected before 2000 and 41 before 2010. Forty-five studies were conducted over 1 to 2 years, and 10 studies involved sampling periods of at least 5 years. Across the 71 studies, 857 measurements were compiled: 128 included both LF and TF fluxes, 588 reported LF fluxes only, 65 reported LF concentrations only, 27 reported TF fluxes only, 2 combined LF concentrations with TF fluxes, and 22 included measurements of open precipitation. MeHg was measured far less frequently with 181 measurements for MeHg in LF and 56 for MeHg TF deposition flux (see Fig. 1, Fig. 2, Table 2).

Most measurements originate from Europe (41% for LF, 38% for TF), the Eastern United States (32% for LF, 43% for TF), and Asia (25% for LF, 17% for TF) (see Fig. 1 and Figures S1–S3). The small number of studies conducted south of 20${}^{\circ }$N or north of 50${}^{\circ }$N prevents the identification of latitudinal trends in LF deposition fluxes or concentrations.

The collected data are primarily situated in temperate (C-climate, 225 measurements) or continental (D-climate, 603 measurements) zones, and mostly under wet precipitation regimes (f-type, 640 measurements). Nonetheless, 18 of the 30 Köppen-Geiger climate classes are represented in the database (see Fig. 1, Fig. 2 and Figures S1-S3).

The dataset is dominated by measurements from deciduous broadleaved forests (296 records, including 226 LF-only) and evergreen needleleaved forests (402 records, including 304 LF-only). Additional measurements are available from tropical raingreen broadleaved forests and mangroves (see Fig. 2). A substantial fraction of the data comes from mixed forests containing both broadleaved and needleleaved species.

### 3.3 Statistical summary of the LF and TF datasets

#### 3.3.1 Summary of LF measurements in the dataset

**Fig. 3:**
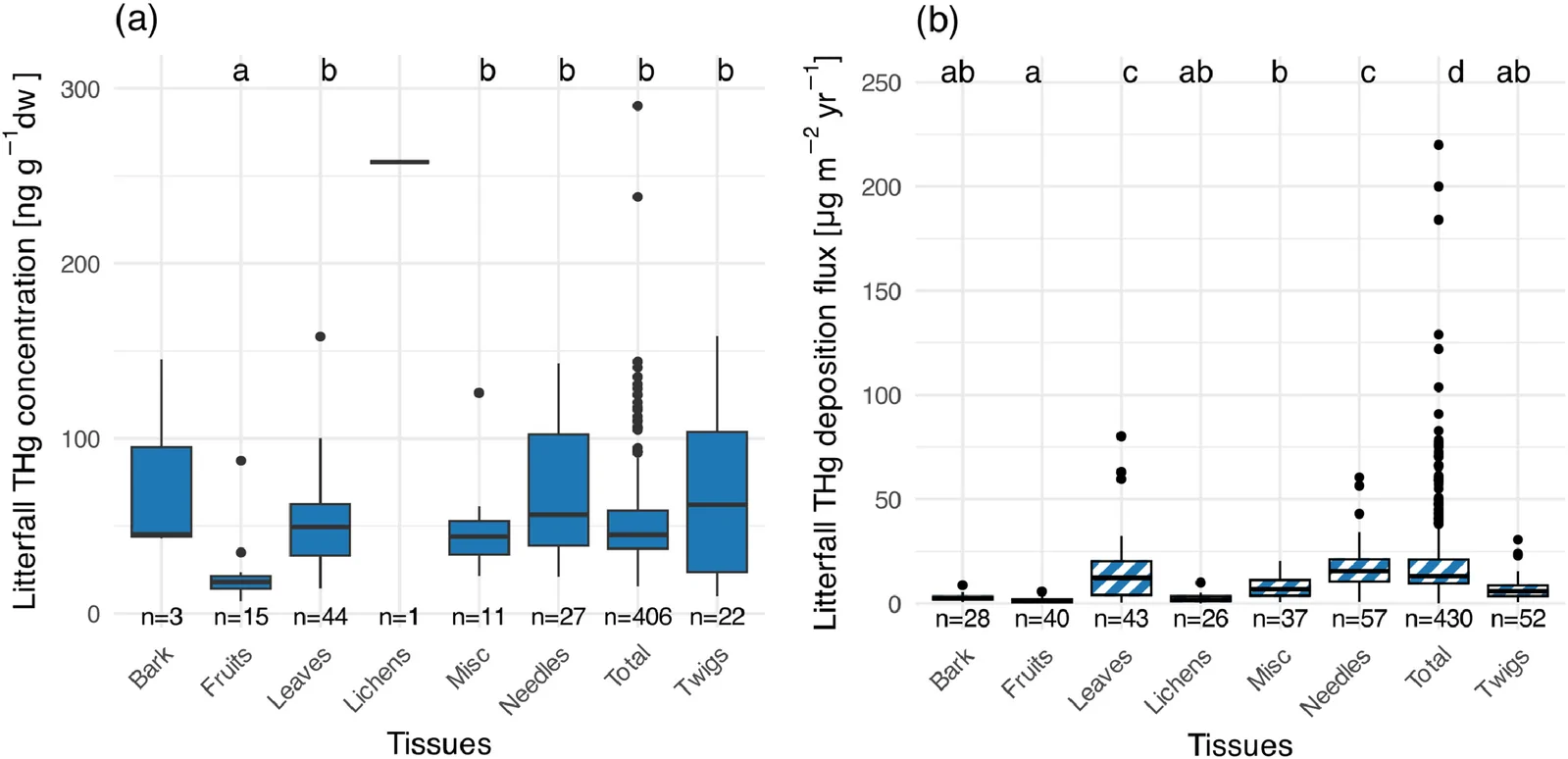
Comparison of (a) litterfall (LF) Hg concentrations in ng g^−1^ dw (dry weight) and (b) fluxes among tissue types. n indicates the number of measurements per tissue. Letters indicate Tukey’s honestly significance difference groupings (α = 0.05), where tissues sharing a letter do not differ significantly.

THg and MeHg concentrations and fluxes in LF and TF exhibited substantial variation across sites and studies. Total-LF THg concentrations range from 15.5 to 290.0 ng g^−1^ dry weight (dw), with a mean (± standard deviation (SD)) of 51.2 ± 25.9 ng g^−1^ dw. Differences among tissues are small: Fruits-LF show the lowest THg concentrations (mean ± SD: 22.4 ± 19.1 ng g^−1^ dw), but no significant differences are found among other tissues or relative to Total-LF Hg concentration (Fig. 3a). Mean ± SD THg concentrations range from 49.3 ± 28.0 ng g^−1^ dw in Misc-LF to 69.5 ± 36.9 ng g^−1^ dw in Needles-LF. Lichens-LF show a higher concentration (258.0 ng g^−1^ dw), but this is based on a single study.

Annual Total-LF THg deposition fluxes range from 0.1 to 219.9 µg m^−2^ yr^−1^, with a mean of 20.5 ± 23.3 µg m^−2^ yr^−1^, and 90% of measurements below 41.8 µg m^−2^ yr^−1^. n. Higher deposition fluxes are observed for Total-LF (mean ± SD: 20.5 ± 23.3 µg m^−2^ yr^−1^), followed by Needles-LF (17.6 ± 11.9 µg m^−2^ yr^−1^), and Leaves-LF (15.8 ± 17.0 µg m^−2^ yr^−1^). Lower fluxes are associated with Fruits-LF (1.7 ± 1.4 µg m^−2^ yr^−1^), Lichens-LF (2.5 ± 2.1 µg m^−2^ yr^−1^), Bark-LF (2.9 ± 1.7 µg m^−2^ yr^−1^), and Twigs-LF (7.2 ± 5.9 µg m^−2^ yr^−1^) (Fig 3b).

Total-LF MeHg concentration range from 0.1 to 0.8 ng g^−1^ dw (mean ± SD: 0.2 ± 0.1 ng g^−1^ dw). Total-LF MeHg deposition fluxes range from 0.0 to 0.6 µg m^−2^ yr^−1^ (0.13 ± 0.13 µg m^−2^ yr^−1^). Because MeHg was measured only in Total-LF, variation among tissue types cannot be assessed.

#### 3.3.2 Summary of TF measurements in the dataset

THg TF concentration range from 3.3 to 9641.5 ng L^−1^ (mean ± SD: 179.6 ± 1099.2 ng L^−1^) with only three measurements exceeding 100.0 ng L^−1^and five measurements exceeding 70.0 ng L^−1^.

THg TF deposition fluxes range from 2.1 to 107.9 µg m^−2^ yr^−1^ and averaged 15.3 ± 13.5 µg m^−2^ yr^−1^. Four measurements are higher than 50.0 µg m^−2^ yr^−1^.

MeHg TF concentrations range from 0.1 to 0.5 ng L^−1^ (mean ±SD: 0.2 ± 0.1 ng L^−1^), with two measurements higher than 0.3 ng L^−1^. MeHg TF deposition fluxes range from 0.0 to 0.6 µg m^−2^ yr^−^(mean ± SD: 0.1 ± 0.1 µg m^−2^ yr^−1^), with four measurements higher than 0.3 µg m^−2^ yr^−1^.

The variability in THg and MeHg TF data does not follow a clear geographic or contamination gradient. Only a few studies (11 measurements from 4 of the 71 studies) explicitly report sampling in contaminated environments but, no further information is available regarding local pollution levels or proximity to anthropogenic source.

### 3.4 Variability in collected THg LF concentrations and deposition fluxes among landcover

**Fig. 4:**
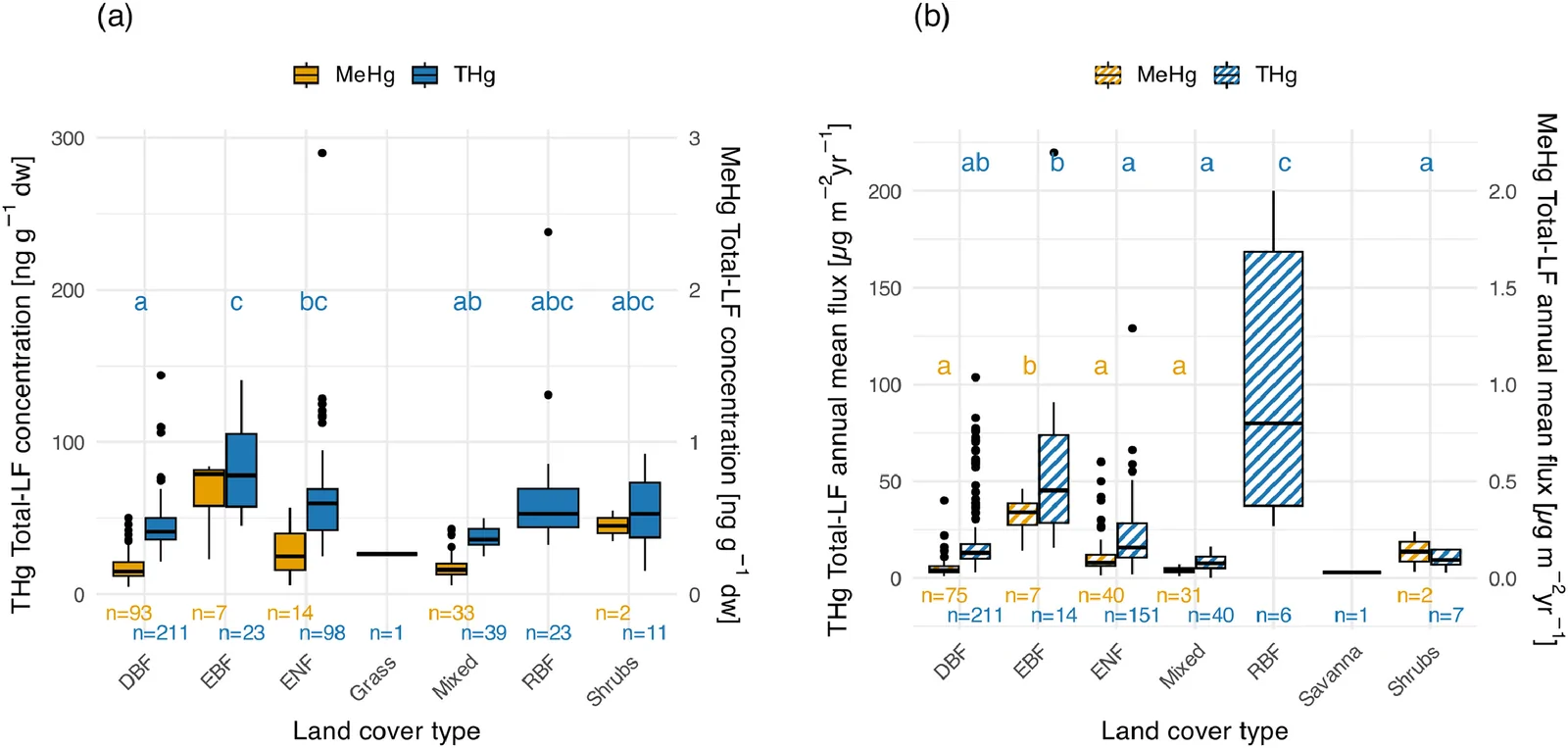
Comparison of Total-litterfall (LF) THg (blue) and MeHg (orange) (a) concentrations in ng g^−1^ dw (dry weight) and (b) fluxes across land-cover types. Land-cover categories include DBF = deciduous broadleaved forest, EBF = evergreen broadleaved forest, ENF = evergreen needleleaved forest, Grass = grassland, Mixed = mixed forest, RBF = raingreen broadleaved forest, Savanna = savanna, and Shrubs= shrubs. The number of measurements per land-cover type is indicated (n). Letters denote Tukey’s honestly significance difference groupings (α = 0.05), where groups sharing a letter do not differ significantly. No letters are shown for MeHg in panel (a) because no significant differences among land-cover types were found.

Total-LF concentrations and fluxes include in the dataset were collected and vary across diverse land-cover types. Total-LF THg concentrations were collected over evergreen broadleaved forests, evergreen needleleaved forests, raingreen broadleaved forests, deciduous broadleaved forests, and shrubs. Both Total-LF THg concentrations and deposition fluxes were significantly affected by land-cover type, whereas MeHg Total-LF concentrations were not. However, MeHg Total-LF deposition fluxes did differ significantly among land-cover types (Fig. 4).

Total-LF THg concentrations and deposition fluxes were highest in evergreen broadleaved forests (respectively mean ± SD: 81.3 ± 27.9 ng g^−1^ dw and 58.1 ± 52.7 µg.m^−2^ yr^−1^, respectively), which also exhibited the highest MeHg deposition fluxes (0.3 ± 0.1 µg.m^−2^ yr^−1^). The lowest Total-LF THg concentrations were observed in deciduous broadleaved and mixed forests (43.8 ± 14.2 and 37.0 ± 6.5 ng g^−1^ dw, respectively, Fig. 4a). Raingreen broadleaved forests displayed the highest Total-LF THg deposition flux (101.1 ± 78.4 µg.m^−2^ yr^−1^, Fig. 4b), although their THg concentrations (65.7 ± 43.2 ng g^−1^ dw) did not differ significantly from those of other land-cover types (Fig. 4a).

### 3.5 Variability in THg LF concentrations and deposition fluxes among climates

**Fig. 5:**
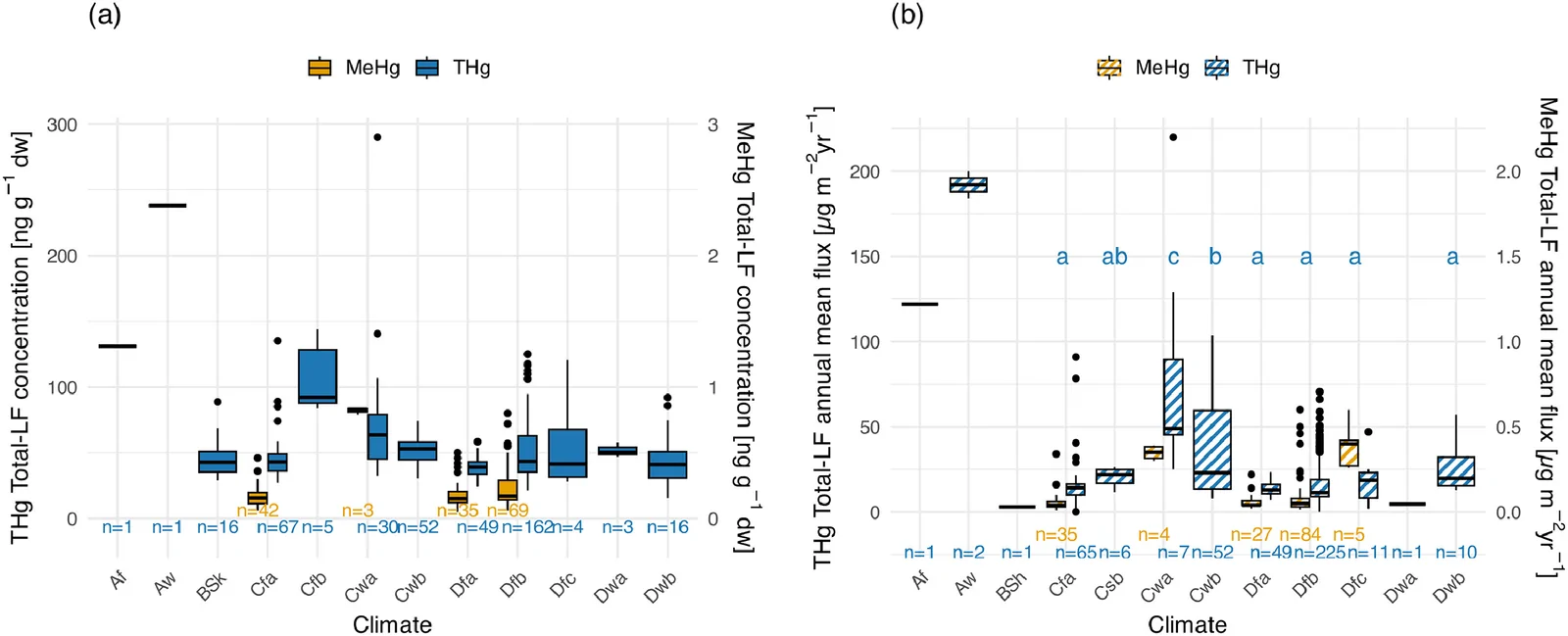
Comparison of Total-litterfall (LF) THg (a) concentrations in ng g^−1^ dw (dry weight) and (b) fluxes across climate types. Climate types include: Af = tropical rainforest, Am = tropical monsoon, BSh = hot semi-arid climate, Cfa = humid subtropical climate, Csb = warm summer Mediterranean climate, Cwa = monsoon influenced humid subtropical climate, Cwb = subtropical highland climate, Dfa = hot summer humid continental climate, Dfb = warm summer humid continental climate, Dfc = subarctic climate, Dwa = monsoon-influenced hot summer humid continental climate, Dwb = monsoon-influenced warm summer humid continental climate. The number of measurements per climate type is indicated (n). Letters denote Tukey’s honestly significance difference groupings (α = 0.05), where groups sharing a letter do not differ significantly. No letters are shown in panel (a) because no significant differences among climate-ere was found.

Variability of Total-LF against climatic factors, –namely the full Köppen Geiger climate code, the main climate group (first letter), and the precipitation regime (second letter) – were evaluated. In the collected dataset, none of these climate descriptors significantly influenced Total-LF THg or Total-LF MeHg concentrations (see Fig. 5a).

By contrast, all climate descriptors significantly affected Total-LF THg fluxes, with R^2^ values for fixed effects of 0.005 (main climate group), 0.07 (precipitation regime), and 0.22 (full climate classification). MeHg fluxes did not show significant differences, likely due to insufficient sample sizes within climate groups. As shown in Fig. 5b, Total-LF THg fluxes were highest under Cwa (monsoon-influenced humid subtropical climate, 80.5 ± 69.9 µg m^−2^ yr^−1^), followed by Cwb (subtropical highland climate, 34.2 ± 25.9 µg m^−2^ yr^−1^) and Csb (warm summer Mediterranean climate, 20.5 ± 5.8 µg m^−2^ yr^−1^).

### 3.6 Variability in THg TF concentrations and deposition fluxes among climate and land cover

The analysis of TF THg was restricted to the land-cover type because the TF dataset was heavily dominated by measurements from Dfb climate (n = 49 for concentrations; n = 106 for fluxes), with few measurements for other climate types (e.g., 8 in Cwa, 7 in Dfc and Dwb). This imbalance prevents a meaningful assessment of climate effects on TF THg. Expanded TF measurements outside humid-continental (Dfb) climates will be required to assess climate-driven variability in TF.

**Fig. 6:**
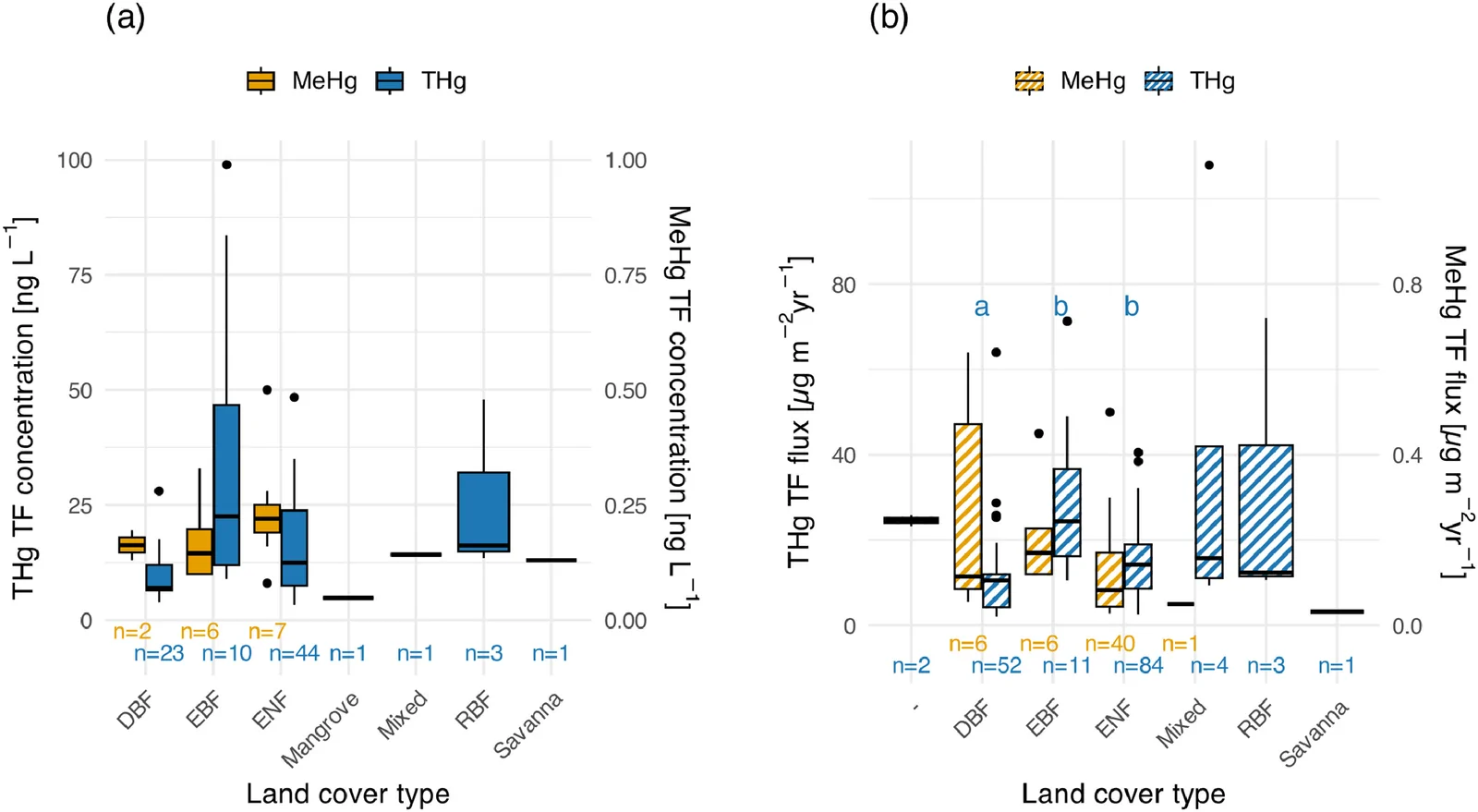
Comparison of throughfall (TF) THg (a) concentrations and (b) fluxes across land-cover types. Land-cover types include: DBF = deciduous broadleaved forest, EBF = evergreen broadleaved forest, ENF = evergreen needleleaved forest, Mangrove = Mangrove, Mixed = mixed forest, RBF = raingreen broadleaved forest, Savanna = savanna. Three outliers with concentrations exceeding 900 ng L^−1^ were removed in panel (a). The number of measurements per land-cover type is indicated (n). Letters denote Tukey’s honestly significance difference groupings (α = 0.05), where groups sharing a letter do not differ significantly.

No significant differences in TF THg concentrations were found among land-cover types, regardless of whether the three outliers (> 900.0 ng L^−1^) were included (Fig. 6a). In contrast, TF THg fluxes differed significantly among land-cover types. Fluxes were highest in evergreen needleleaved and broadleaved forests (14.6 ± 7.8 µg m^−^² yr^−^¹ and 29.1 ± 18.7 µg m^−^² yr^−^¹, respectively) and lowest in deciduous broadleaved forests (10.8 ± 9.7 µg m^−^² yr^−^¹; Fig. 6b).

## 4 Example of Database Use

To illustrate the dataset’s utility, two relative indicators are presented: (i) the LF/TF ratio, which reflects the proportion of THg absorbed by foliage compared to that deposited on and washed off leaves, and (ii) the TF/open precipitation ratio, which quantifies the fraction of THg intercepted by the canopy and subsequently washed off. Together, these indicators offer complementary insights into bioclimatic variability and canopy-related processes, particularly in contexts where concurrent atmospheric Hg measurements are unavailable.

### 4.1 Bioclimatic controls on LF/TF ratios

**Fig. 7:**
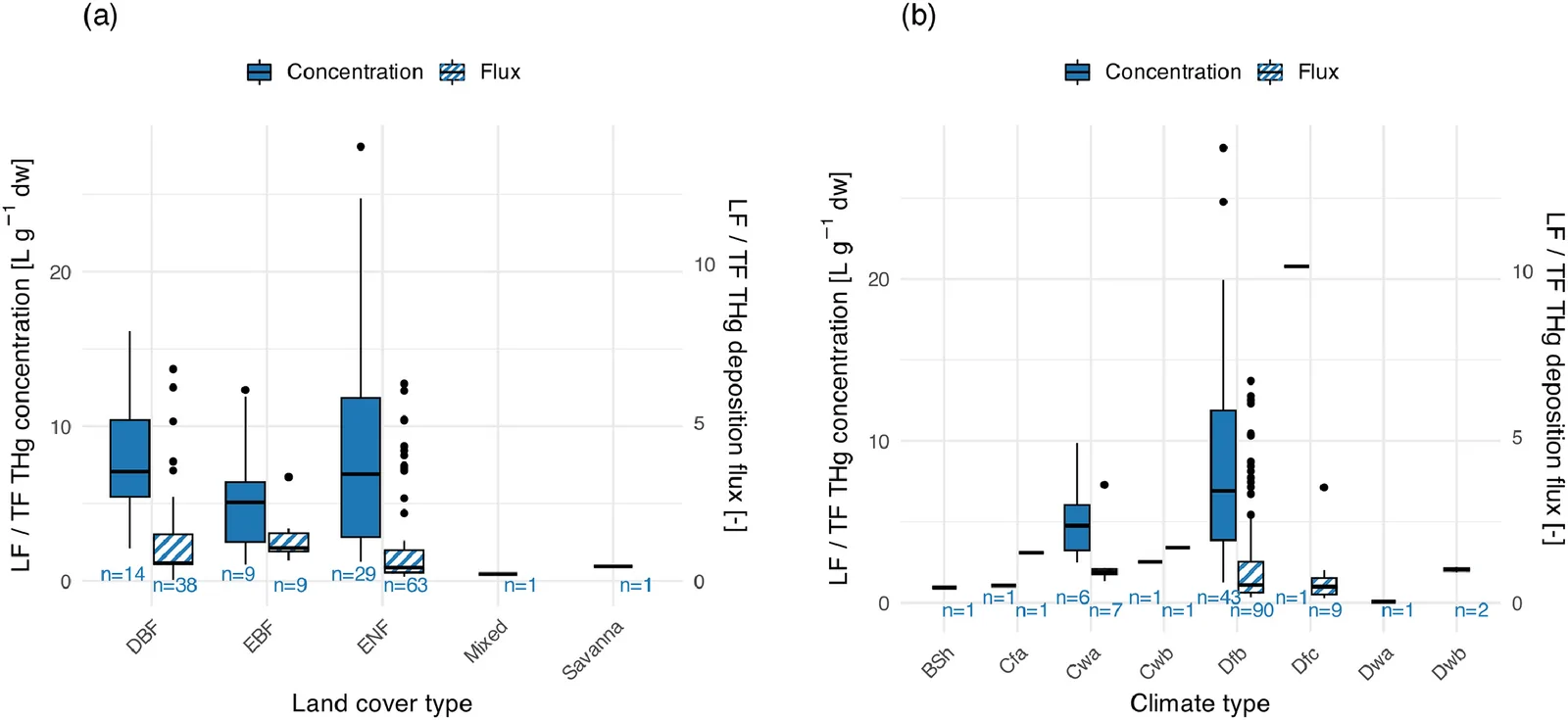
Litterfall (LF) to throughfall (TF) ratios of THg concentrations L g^−1^ dw (dry weight) and fluxes across (a) land-cover type and (b) climate types. Only total-litterfall measurements were used to calculate ratios. Land-cover types: DBF = deciduous broadleaved forest, EBF = evergreen broadleaved forest, ENF = evergreen needleleaved forest, Grass = grassland, Mixed = mixed forest, RBF = raingreen broadleaved forest, Savanna= savanna, and Shrubs= shrubs. Climate types: BSh = hot semi-arid climate, Cfa = humid subtropical climate, Cwa = monsoon-influenced humid subtropical climate, Cwb = subtropical highland climate, Dfb = warm summer humid continental climate, Dfc = subarctic climate, Dwa = monsoon-influenced hot summer humid continental climate, Dwb = monsoon-influenced warm summer humid continental climate. The number of measurements per land-cover and climate types is shown (n).

The LF/TF ratios represent the balance between vegetation uptake and canopy wash-off of THg varies across land-cover types. This ratio serves as a simple indicator of canopy-mediated partitioning of atmospheric THg and is less sensitive to fluctuations in atmospheric Hg concentrations than LF or TF values considered independently. No significant differences in LF/TF ratios were detected, either for concentrations or for fluxes, across land-cover types (see Fig. 7a) or climate categories (see Fig. 7b), with mean (± SD) values of 8.1 (± 6.1) L.g^−1^ dw for concentration ratios and 2.5 (± 3.2) L.g^−1^ dw for flux ratios.

The consistent LF/TF ratios across biomes suggest that THg partitioning between vegetation uptake (LF) and canopy deposition (TF) is driven by similar underlying processes.

### 4.2 Bioclimatic controls on TF/ open-precipitation

**Fig. 8:**
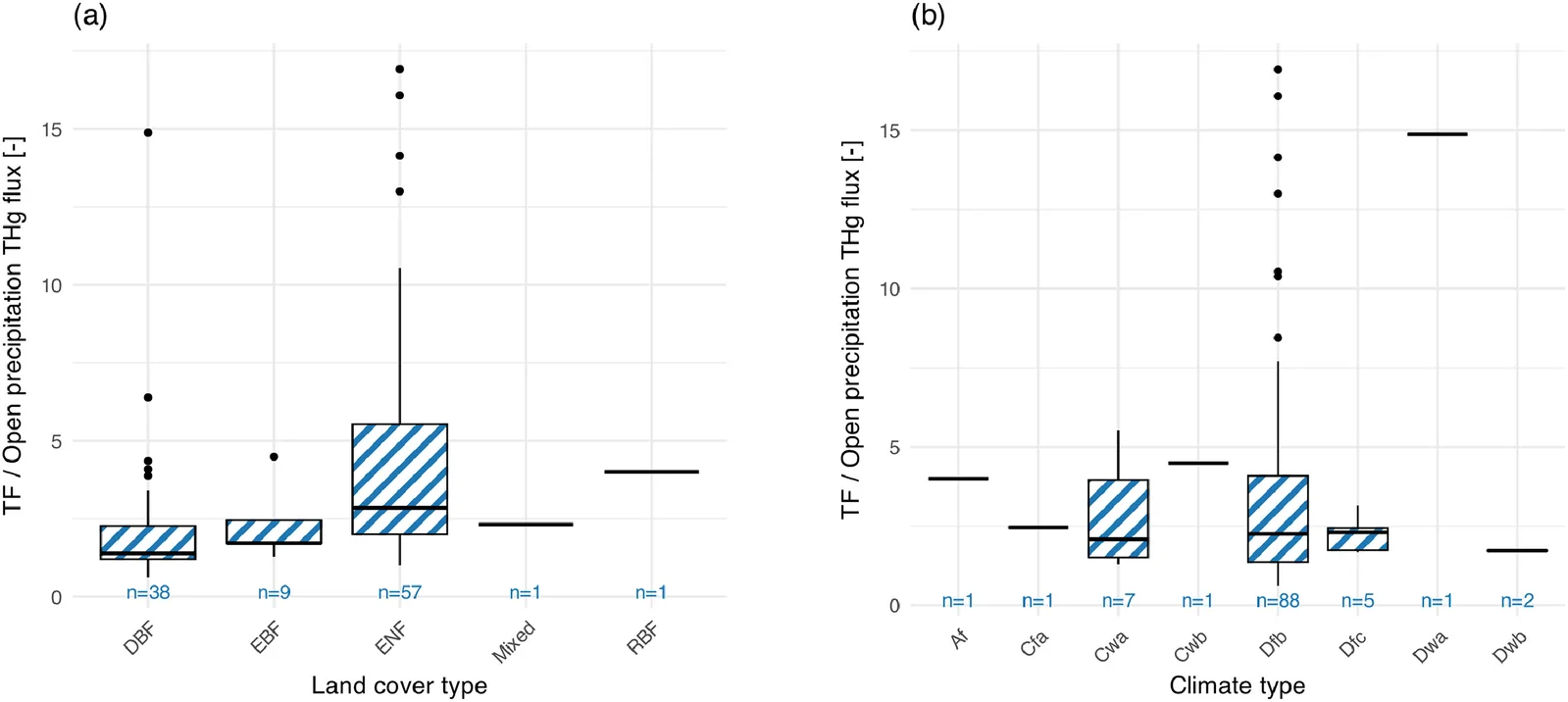
Throughfall to open-precipitation ratios of THg concentrations and fluxes across (a) land-cover and (b) climate types. Land-cover types: DBF = deciduous broadleaved forest, EBF = evergreen broadleaved forest, ENF = evergreen needleleaved forest, Grass = grassland, Mixed = mixed forest, RBF = raingreen broadleaved forest, Savanna= savanna, and Shrubs= shrubs. Climate types: BSh = hot semi-arid climate, Cfa = humid subtropical climate, Cwa = monsoon-influenced humid subtropical climate, Cwb = subtropical highland climate, Dfb = warm summer humid continental climate, Dfc = subarctic climate, Dwa = monsoon-influenced hot summer humid continental climate, Dwb = monsoon-influenced warm summer humid continental climate. The number of measurements per land-cover and climate types is shown (n).

The TF/open precipitation ratios represent the amount of THg retained by the canopy and subsequently washed off as TF. Because open precipitation concentrations were measured only infrequently (see Table 2 and Fig. 2), TF/open precipitation ratios were limited to flux ratios. TF/open precipitation ratios varied widely, ranging from 0.6 to 73.4, with 90% of values below 7.6. Yet, no significant differences were detected in these ratios across land-cover types (see Fig. 8a) or climate categories (see Fig. 8b), with mean (±SD) values of 4.7 (± 9.4).

However, the scarcity of open precipitation concentration data prevents interpretation of these patterns.

## 5 Experimental Design, Materials and Methods

### 5.1 Literature screening

**Fig. 9:**
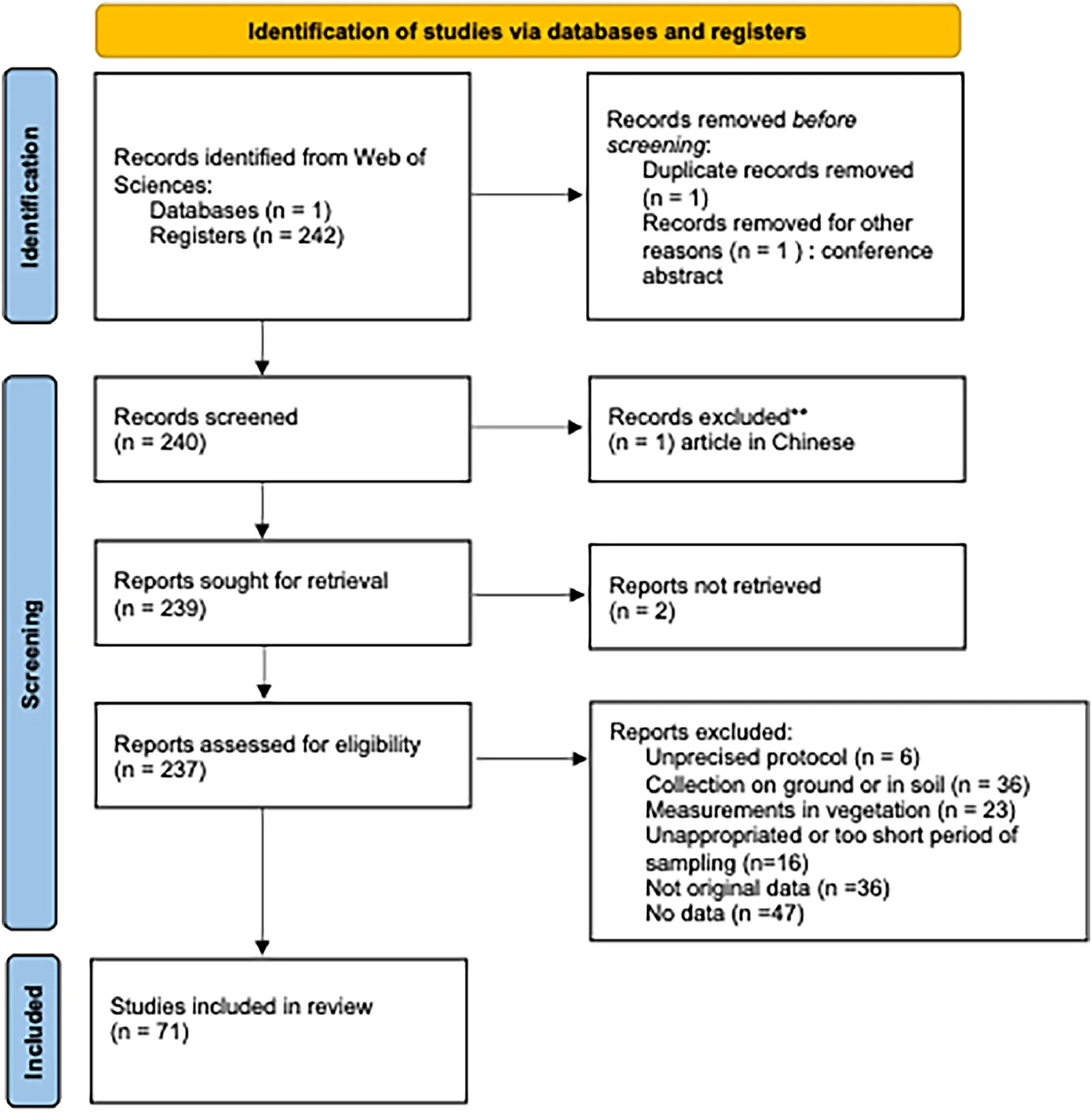
PRISMA flow diagram illustrating the construction process of the MFallDa database.

Literature screening was conducted on October 6th, 2025 on the Web of Science Core Collection using the topic search “mercury OR Hg” AND “litterfall OR throughfall”, allowing us to simultaneously collect studies with THg or MeHg data. In total, 242 publications with measurement-based Hg LF or TF data published between 1987 and 2024 were retrieved, as shown in Fig. 9. Out of these 242 publications, two conference papers and one manuscript that could not be retrieved were excluded. This list of publications is available in the file WOS_search.csv (see Sect. 3).

From this initial list of 237 publications, only publications in which LF was collected using baskets or traps were collected. Studies where litter was collected directly from the ground (as litter degradation may have affected Hg concentrations [11]) or directly from trees (as Hg accumulation occurs progressively throughout the year [12]) were excluded. For example, some data for the Amazon rainforest in Feinberg et al. (2022) [13] were excluded from our dataset for these reasons. A few publications were also excluded due to very short (e.g., days to weeks) or non-relevant periods (e.g., April–October in the Northern Hemisphere [14]), which cannot be reliably extrapolated to annual fluxes without knowledge of the local litterfall regime. After screening, 71 publications with original measurements remained and were used to create MFallDa, see Fig. 9.

To ensure the representativity of our literature collection, the selected list was compared with the dataset of Feinberg et al. (2022) [13], who conducted a broader search using “mercury litter*”, including studies reporting litter fluxes rather than explicitly using the term “litterfall”, but limited to the 2016–2020 period. Feinberg et al. (2022), identified 334 publications for this period, of which only 10 were retained. By limiting screening to roughly halve the number of publications while covering a 30-year period instead of four years, some articles were inevitably missed; however, only 3 [[15], [16], [17]] of these would have met MFallDa collection criteria. This comparison confirms that the dataset provided here is a representative overview of the available LF Hg measurements while maintaining a manageable screening effort over an extended temporal coverage.

### 5.2 Database construction from publications

Data gathered from the publications included data of LF and TF THg and MeHg concentrations and fluxes, associated vegetation cover, dominant plant species, and land cover type (evergreen broadleaved forest, evergreen needleleaved forest, raingreen broadleaved forest, deciduous broadleaved forest, mixed forests, shrublands, grasslands), as well as LF biomass, annual rainfall and TF depth, and open wet THg and MeHg deposition concentrations and fluxes when available. Methods of LF and TF collection were also recorded.

Data on a yearly basis and specific to plant cover were collected, excluding multi-year or cross-plant-type averages when individual measurements were provided. When LF was sorted during collection, details about LF tissues (leaves, fruits, barks, etc.) were included; however, unsorted LF collections (hereafter referred to as “Total-LF”) dominated tissue-separated measurements, representing over 65% of LF measurements.

For publications reporting ecosystem manipulation experiments (e.g., litter addition or precipitation removing), only control data were collected. In addition, geographic coordinates, altitude, year of study, and Köppen-Geiger climate classification were included. This classification defines climate type based on threshold values and seasonality of monthly air temperature and precipitation [18,19]. Major climate types are indicated by the first letter, the second letter reflects seasonal precipitation patterns, and the third letter represents temperature characteristics. Climate classification was extracted from a 0.0083${}^{\circ }$ (∼1-km) resolution map for the 1991–2000 period [20] and then converted to a 0.01${}^{\circ }$ resolution to match the longitude and latitude coordinates of the constructed database. For the five measurements (two locations) without available climate data in the 0.0083${}^{\circ }$ map, 0.5 ${}^{\circ }$ and 1${}^{\circ }$ resolution maps from the same source [20].

### 5.3 Database updating procedure

To ensure the MFallDa remains robust and useful, it is essential to maintain regular updates and follow harmonized sampling protocols. LF and TF measurements should capture annual variability, with litter collected progressively as it naturally falls rather than directly from trees, and include leaves, twigs, and other plant parts, to accurately reflect THg and MeHg accumulation over time.

Users are encouraged to propose modifications to the MFallDa or documentation via GitHub (https://github.com/IGE-mercury/MFallDa/). The required contribution workflow is explained in the README.md file. Contributors who wish to update the database should first create their own copy of the repository available on GitHub (create a *fork*), make their changes in that copy, and then submit the changes for review (submit a *pull* request against the *dev* branch). Once the changes are approved by the repository owner, they will be merged into the main working version of the database. A new release incorporating these updates will then be created on Zenodo. For users who do not use GitHub, data submissions by email at mfallda@univ-grenoble-alpes.fr are also welcome.

### 5.4 Collected data environmental variability

LF and TF THg and MeHg concentrations and deposition fluxes variation across bioclimatic factors, including land cover type, overall climate classification, major climate type, and precipitation regime (section 3.3 to 3.6) are presented. In complement, an application of the database is proposed with the LF/TF and TF/open precipitation ratios description across bioclimatic factors, including land cover type, overall climate classification, major climate type, and precipitation regime (section 4). Only groups with more than five measurements were included in this descriptive analyse. To account for the non-independence of measurements originating from the same study, a linear mixed-effects model with each bioclimatic factor as a fixed effect and study identity as a random effect was used. Pairwise comparisons between levels of each factor were conducted using Tukey’s adjustment for multiple testing, with a significance threshold of 0.05.

## Limitations

Our dataset covers multiple regions across all continents except Oceania and Antarctica, encompassing 18 different climate types. However, measurements from North America, Europe, and China dominate, while data from South America and Africa remain underrepresented, despite the key role of the Amazonian forest in vegetation-mediated fluxes [21]. Similarly, evergreen and needle leaved forests measurements dominate in the data set, limiting the statistical power of land cover effect analysis. Besides, only a few studies (11 measurements of TF from 4 of the 71 studies) explicitly report sampling in contaminated environments while no further information is available regarding local pollution levels or proximity to anthropogenic source.

## Ethics Statement

The authors have read and follow the ethical requirements for publication in Data in Brief and confirm that the current work does not involve human subjects, animal experiments, or any data collected from social media platforms.

## CREDIT Author Statement

Laura Sereni: conceptualization, data curation, formal analysis, writing original draft, review, and editing. Erfan Jahangir: resources (created and managed the infrastructure hosting the database (GitHub and Zenodo)). Xinbin Feng, Ivan J. Fernandez, Anne-Hélène Fostier, David Grigal, Antía Gómez-Armesto, Randy Kolka, John Munthe, Tomáš Navrátil, Juan Carlos Nóvoa-Muñoz, Andrea Parente-Sendín, Xabier Pontevedra-Pombal, Emmanoel Vieira Silva-Filho: Resources (provided data), Hélène Angot: conceptualization, supervision, funding acquisition, reviewing, and editing.

## Declaration of Generative AI and AI-assisted Technologies in the Manuscript Preparation Process

During the preparation of this work, the authors used MISTRAL/AI for language polishing (grammar and clarity improvements). No content generation, restructuring of scientific arguments, or addition of scientific interpretation was performed by AI. All scientific content and interpretation were developed by the authors, and the manuscript has been revised and edited as needed to ensure consistency with journal standards of originality and author voice. The authors take full responsibility for the content of the published article.

## Data Availability

ZenodoMFallDa: Data and code accompanying the data paper (Original data)

## Appendix Supplementary Materials

Image, application 1
