# Supplementary material for: Standardizing mercury litterfall and throughfall data for comparative research: Introducing the MFallDa global database
Source: Data Brief. 2026 Aug 27;68:113206. doi: 10.1016/j.dib.2026.113206 (PMC13579965; doi:10.1016/j.dib.2026.113206)
Supplement: Supplementary file 1 [file mmc1.docx]

Supplementary figures

Figure S1: Measurements sites and number of measurements in the collected dataset, shown by measurement type and main climate category over Asia for (a) litterfall and (b) throughfall. Climate categories: Af = tropical rainforest climate, Am = Tropical monsoon climate, Aw = tropical savanna climate, BSh = Hot semi-arid climate, BSk = Cold semi-arid climate, Cfa = humid subtropical climate, Cwa = Monsoon-influenced subtropical climate, Cwb = Subtropical highland climate, Dfa = Hot summer humid continental climate, Dfb = Warm summer humid continental climate, Dfc = Subarctic climate, Dwa = Monsoon-influenced hot summer humid continental climate, Dwb = Monsoon-influenced warm summer humid continental climate, Dwc = Monsson-influenced subarctic climate, and ET = tundra climate.

Figure S2: Measurements sites and number of measurements in the collected dataset, shown by measurement type and main climate category over Europe for (a) litterfall and (b) throughfall. Climate categories: BSk = Cold semi-arid climate, Cfa = humid subtropical climate, Cfb = Temperate oceanic climate, Cfc = Subpolar oceanic climate, Csa = Hot summer Mediterranean climate, Csb = Warm summer Mediterranean climate, Dfa = Hot summer humid continental climate, Dfb = warm summer humid continental climate, Dfc = subarctic climate, Dfd = Extremely cold subarctic climate, and ET= tundra climate.

Figure S3: Measurements sites and number of measurements in the collected dataset, shown by measurement type and main climate category over North America for (a) litterfall and (b) throughfall. Climate categories: Am = Tropical monsoon climate, Aw = Tropical savanna, BSh = Hot semi-arid climate, Cfa = Humid subtropical climate, Cfb = Temperate oceanic climate, Dfa = Hot summer humid continental climate, Dfb = Warm summer humid continental climate, Dfc = Subarctic climate, Dsc = Mediterranean-influenced subarctic climate, Dwc = Monsson-influenced subarctic climate, and ET = tundra climate.
